# Deep Compressed Sensing for Learning Submodular Functions

**DOI:** 10.3390/s20092591

**Published:** 2020-05-02

**Authors:** Yu-Chung Tsai, Kuo-Shih Tseng

**Affiliations:** Department of Mathematics, National Central University, Taoyuan City 32001, Taiwan; 107221020@cc.ncu.edu.tw

**Keywords:** submodularity, compressed sensing, autoencoder, deep learning

## Abstract

The AI community has been paying attention to submodular functions due to their various applications (e.g., target search and 3D mapping). Learning submodular functions is a challenge since the number of a function’s outcomes of N sets is $2^{N}$. The state-of-the-art approach is based on compressed sensing techniques, which are to learn submodular functions in the Fourier domain and then recover the submodular functions in the spatial domain. However, the number of Fourier bases is relevant to the number of sets’ sensing overlapping. To overcome this issue, this research proposed a submodular deep compressed sensing (SDCS) approach to learning submodular functions. The algorithm consists of learning autoencoder networks and Fourier coefficients. The learned networks can be applied to predict $2^{N}$ values of submodular functions. Experiments conducted with this approach demonstrate that the algorithm is more efficient than the benchmark approach.

## 1. Introduction

**Figure 1:**
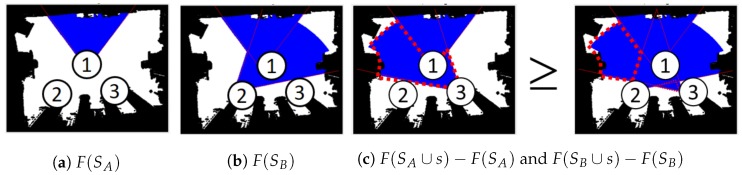
Illustration of submodularity. The decimal number represents the selected sensor. The blue and white colors represent the covered and uncovered areas, respectively. (**a**) $F(S_{A})$ represents the area covered by $S_{A}$, where $S_{A}=\{1\}$. (**b**) $F(S_{B})$ represents the area covered by $S_{B}$, where $S_{B}=\{1,2\}$. (**c**) The red dash lines represent the submodular gain after adding *s*, where s={3}. The left figure shows the $F\left(S_{A}\cup s\right)-F\left(S_{A}\right)$ and the right figure shows that $F\left(S_{B}\cup s\right)-F\left(S_{B}\right)$.

AI and robotics communities have been paying more attention to submodularity (see Definition 1 and Figure 1) due to its variant applications (e.g., information collection [1], task assignment [2], and target search [3]) and theoretical guarantees of solutions. The advantage of formulating a problem as maximizing submodular functions is that greedy algorithms can give theoretical guarantees under cardinality [4], knapsack [5], and routing constraints [6]. Moreover, if the objective function is under the cardinality constraint, greedy algorithms can generate solutions over (1−1/e) of the optimum [4]. Unless P=NP, no polynomial-time algorithms can outperform greedy approaches [7].

**Definition** **1.** (Submodularity (Nemhauser et al., 1978)). *Given a finite set S = {1,2,…,N}, a submodular function is a set function $F:2^{N}\to R$ which satisfies the diminishing return property. For every $S_{A},S_{B}\subseteq S$ with $S_{A}\subseteq S_{B}$ and every s⊆S, $F\left(S_{A}\cup s\right)-F\left(S_{A}\right)\geq F\left(S_{B}\cup s\right)-F\left(S_{B}\right)$ holds.*

To illustrate the concept of submodularity, an example is shown in Figure 1. There are three ground sets (S={1,2,3}). $S_{A}=\left\{1\right\}$ and $S_{B}=\left\{1,2\right\}$ represent the selected two sets, respectively. The set $S_{B}=\left\{1,2\right\}$ means that the sensors are selected at location 1 and 2. $F(S_{A})$ and $F(S_{B})$ mean the coverage of sensor at location 1 and {1,2} (see Figure 1a,b), respectively. The submodular gain of $S_{A}$ and $S_{B}$ after adding a set s={3} is represented by the red dashing lines (see Figure 1c). It is obvious that the coverage function satisfies the diminishing return property. In other words, the objective function of maximizing coverage is submodular. Greedy approaches can generate near-optimal solutions even if this is an NP-hard problem [4,7].

Although submodularity sheds light on solving NP-hard problems with theoretical guarantees, the submodular functions are unknown for most of the applications. Therefore, submodular functions need to be learned via real-time sensor data. Learning submodular functions is a challenging problem in machine learning, since there are $2^{N}$ values based on binary values of *N* discrete sets. In [8], the authors proposed Fourier sparse set functions (FSS) to learn submodular functions using compressed sensing techniques [9]. However, the number of Fourier bases is still $2^{N}$. In [3], the authors found the relationship between the set configuration and sparsity of Fourier bases. If there is no sensing overlapping between two sets, the value of its corresponding Fourier basis is zero. Hence, the number of Fourier bases could be dramatically reduced if there are a few overlapping sets. Then, the compressed sensing is feasible for learning submodular functions.

The disadvantages of the spatial Fourier sparse set (SFSS) [3] approach are as follows: First, the precomputation step for computing sets’ sensing overlapping could be time-consuming. Second, the number of bases is relevant to the number of sets’ sensing overlapping. If most of the sets are overlapping, the number of the Fourier basis is close to $2^{N}$. Third, SFSS adopts Hadamard transform, which has Fourier bases consisting of +1 and −1. This transform limits the possibility of Fourier support. These shortages are based on the Hadamard transform which is a linear transform. Deep neural networks could solve these issues through nonlinear transforms.

**Figure 2:**
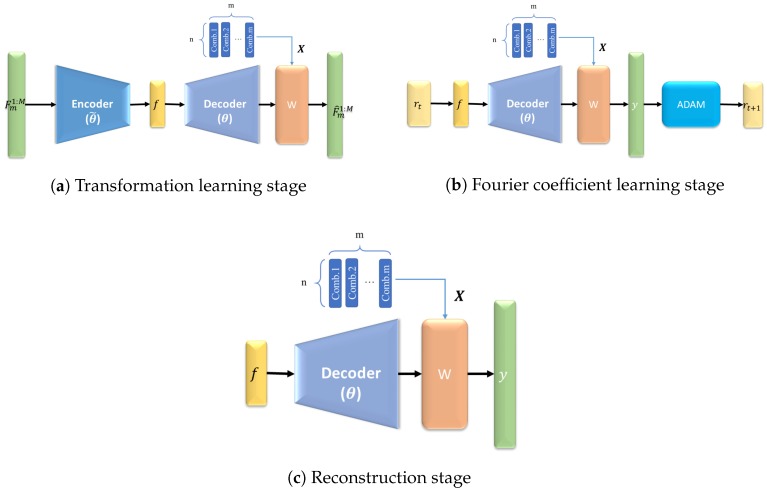
The illustration of the deep neural networks for submodular functions. (**a**) Transformation learning stage: In this stage, the goal is to train the decoder networks through input signal $\left(F_{m}^{1:M}\right)$. The input signal $F_{m}^{1:M}$ is compressed as the Fourier coefficients (f) by encoder networks. Then, the *f* is decompressed by decoder networks. The decompressed *f* and combinational inputs generate the estimated signal $\left({\hat{F}}_{m}^{1:M}\right)$. (**b**) Fourier coefficient learning stage: In this stage, the goal is to train the Fourier coefficients (f) The Fourier coefficients $\left(r_{t}\right)$ at time t are decompressed by the decoder networks. The decompressed *f* and combinational inputs generate the estimated signal *y*. Then, the *y* is computed through *ADAM* to get the Fourier coefficients $\left(r_{t+1}\right)$ at time t+1. (**c**) Reconstruction stage: In this stage, the goal is to predict submodular functions according to f and combinational input.

This research proposes a submodular deep compressed sensing (SDCS) approach to learn submodular functions. The proposed approach consists of three stages (see Figure 2). The transformation learning stage is to learn the nonlinear transform (Θ) via an autoencoder (see Figure 2a), which transfers the data to the Fourier domain. The Fourier coefficient learning stage is to learn the coefficients (f) in the Fourier domain (see Figure 2b). The reconstruction stage is to predict submodular functions according to any combinational data and the Fourier coefficients (f) (see Figure 2c).

The contributions of this paper are as follows: First, the proposed algorithm can learn submodular functions via deep compressed sensing techniques. To the best of our knowledge, this is the first work to learn submodular functions via deep compressed sensing. Second, the nonlinear transformation network is learned for training data. In other words, the algorithm teaches the decoder networks from data to generate sparse coefficients (f) in the frequency domain. Third, the experiments demonstrate that the proposed algorithm is able to reconstruct submodular functions with fewer Fourier bases than the benchmark approach [3].

The paper is organized as follows. Section 2 describes the related work. Section 3 introduces the problem formulation and the proposed deep compressed sensing. Section 4 describes the proposed algorithm. Section 5 describes the experiments. Finally, Section 6 concludes the paper with a summary of the work.

## 2. Relevant Work

This section reviews the prior work of sparse regression, learning submodularity, and deep compressed sensing.

### 2.1. Sparse Regression

Due to “Big Data” applications, scientists need to analyze millions or billions of data points via machine learning techniques. If the data has the sparse property, it can be solved efficiently. The objective function includes loss and regularization terms. The loss term is to minimize the error of estimated data while the regularization term is to avoid overfitting (e.g., L2 norm) or ill-posed problems (e.g., L1 norm). Different norms can provide different regularization purposes. For example, L1-norm was proposed to learn from the data with sparse solutions [10]. Elastic net was proposed to learn from the data and trade-off between L1 and L2 norms [11]. Pairwise elastic net (PEN) was proposed to learn from the data with group sparsity (so called group LASSO) [12,13]. Graphical LASSO was proposed to learn the conditional correlations between high dimensional variables [14].

Sparse regression not only provides rigid theoretical proofs but also supports various applications. For example, the compressed sensing technique is to learn from the data in a certain domain and reconstruct the data in the time or spatial domain with random sampling data [15,16]. Elastic net was applied to select sensors for maximizing the environmental coverage [17]. Graphical LASSO was applied to analyze human operators’ search behavior [18,19].

### 2.2. Submodularity

The set function is submodular if it satisfies diminishing returns property. In [4], the authors show that if the objective function is submodular, a greedy policy finds the solution of maximizing the objective function with theoretical guarantees. In [7], the authors further proved that greedy algorithms give near-optimal guarantees. The applications include sensor placement for indoor temperature prediction [20], motion planning for collecting lake information using multiple robots [21], and collecting WiFi information using UAVs [1].

### 2.3. Learning Submodular Functions

For searching and mapping applications, submodular functions are unknown. The agents need to learn submodular functions via real-time measurements. In [22], the authors proposed probably mostly approximately correct (PMAC) learning, which approximates submodular functions through linear classifiers. However, this approach cannot approximate submodular functions accurately within polynomial samples. In [8], the authors proposed a compressed sensing technique, which can learn submodular functions in the Fourier domain and reconstruct them in the spatial domain. Unfortunately, the number of Fourier bases is still $2^{N}$. In [3], the authors proved that the submodular function is sparse in the Fourier domain when the sets’ sensing is not overlapping in the spatial domain. In other words, the number of the Fourier basis could be polynomial if the sets were spread out. Hence, this approach shows that it is possible to learn submodular functions under certain spatial conditions.

### 2.4. Deep Compressed Sensing

Inspired by the successful applications of deep networks, several deep compressed sensing (DCS) algorithms have recently been proposed. Those approaches can be divided into three classes. The first one is a network-based approach. In [23], the DCS reconstructs the original signal with blocks by a deep fully connected neural network. In [24], the authors apply a stacked denoising auto-encoder (SDA) with a deep fully connected network to learn the representation from training data and to reconstruct test data from their CS measurements. In [25,26,27,28,29], the authors propose convolutional architectures for image reconstruction from low-dimensional measurements. The second one is the frame-based approach. The DCS learns the parameters of the iterative soft thresholding algorithm (ISTA), the parameters of the encoder and layer-dependent threshold [30], or the step size of each layer [31]. The third one is a combination of two classes. In [32], the authors proposed ISTA-Net, which utilizes the advantages of network-based and optimization approaches to design a learnable deep network framework. Instead of handcrafting, the parameters of the autoencoder and networks are learned through the ISTA-Net. This approach inspired us to study whether there are nonlinear transformations which can compress submodular functions into the Fourier domain.

## 3. Problem Formulation

In this section, how to learn submodular functions via compressed sensing (e.g., FSS and SFSS) is introduced [3]. The properties and disadvantages of SFSS are also highlighted. Then, the proposed deep compressed sensing framework to learn submodular functions is introduced.

### 3.1. Learning Submodular Functions via Compressed Sensing

The major difference between compressed sensing techniques for image data and submodular functions is that the size of image data is fixed while the size of submodular functions is $2^{N}$, where N is the number of the sets. Hence, the challenge of learning submodular functions is that the number of a function’s outcomes for *N* sets is $2^{N}$. In [8], the authors first proposed Fourier sparse set (FSS) to learn submodular function using compressed sensing techniques [15,16].

**Figure 3:**
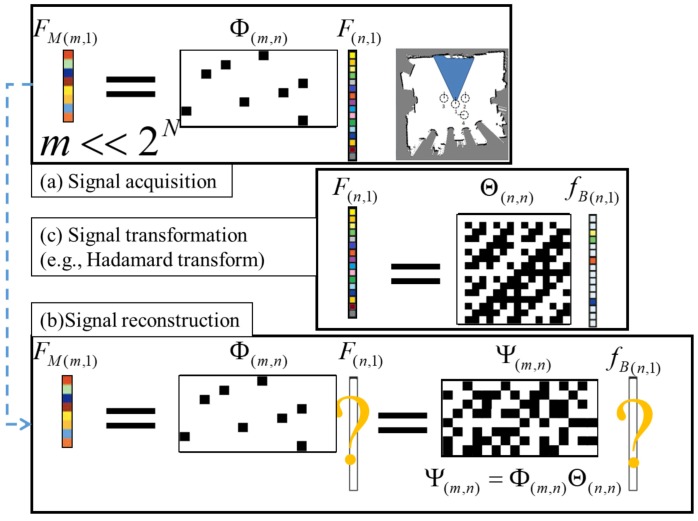
Illustration of the compressed sensing concept [3]. (**a**) $F_{M(m,1)}$ is collected by the system after taking measurements from a signal $F_{\left(n,1\right)}$. The color cells represent real values and black/white cells represent binary values (0 and 1 in Φ while 1 and −1 in Θ.) (**b**) The system has $F_{M(m,1)}$ and tries to recover $F_{\left(n,1\right)}$. (**c**) The signal *F* is sparse in the Fourier domain. In this example, *m* is 8, *n* is 16 and *k* is 4. Given $\Phi_{m,n}$ and $F_{M(m,1)}$, it’s impossible to recover $F_{\left(n,1\right)}$ (m<n). But, given $\Psi_{\left(m,n\right)}$ and $F_{M(m,1)}$, $f_{B}\left(n,1\right)$ can be recovered (k<m).

As Figure 3a shows, assume there are *N* sets and the submodular function is $F_{\left(n,1\right)}$, where $n=2^{N}$. The system first acquires a signal from $F_{\left(n,1\right)}$ via a sensing matrix $\Phi_{\left(m,n\right)}$ and collects $F_{M(m,1)}$ for learning, where m<<n. The system has to recover the signal *F* (see Figure 3b). Notice that this is an ill-conditioned linear inverse problem. However, if the signal is sparse in certain domains, the system can recover *F* via sparse regression [10,33]. As Figure 3c shows, *F* is the inner product of the transform matrix $\Theta_{\left(n,n\right)}$ (e.g., Fourier transform) and coefficient $f_{B(n,1)}$. The $f_{B(n,1)}$ has only *k* nonzero values (so called k-support). Since Θ and Φ are known, the reconstruction matrix Ψ can be computed. Although directly recovering *F* is impossible, the robot can recover $f_{B(n,1)}$ if k<m, and then reconstruct *F*. The signal recovery formulation is given as:

$${\hat{f}}_{B}=\arg\min_{f_{B}}\frac{1}{2}\left|\right|F_{M}-\Psi f_{B}{\left|\right|}^{2}+\lambda ||f_{B}{\left|\right|}_{1}$$

where $f_{B}$ is the submodular function in the Fourier domain, $F_{M}$ is a measurement vector of the submodular function, Ψ is a reconstruction matrix (so called dictionary), Ψ=ΦΘ, and Φ is a sensing matrix and Θ is a Fourier transform matrix.

However, two combinatorial explosion issues of compressed sensing are as follows: First, since the size of submodular functions is $2^{N}$, it is infeasible to access the whole dataset. Second, since the size of the Fourier transform matrix is $2^{N}$ by $2^{N}$, it is infeasible to compute all of the spectrum. In [3], the authors found that if there is no sensing overlap between two sets, the coefficients of its corresponding Fourier basis are zero. This approach is called spatial Fourier sparse set (SFSS). The SFSS approach utilizes the sparsity of submodular functions in the Fourier domain to avoid combinatorial explosion issues.

**Figure 4:**
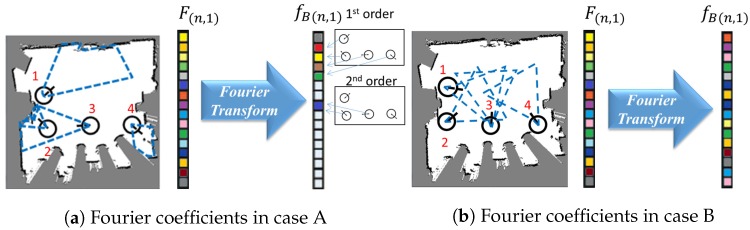
Illustration of the sparsity of submodular functions in the Fourier domain. The black and white areas are obstacles and unoccupied grids, respectively. The black circles and lines represent the robot position and heading, respectively. The blue dash lines are the covering area of the corresponding robot position. The colorful and white cells in the bars represent non-zero and zero values, respectively.

As Figure 4 shows, there are two cases of set configurations. There are four sets (e.g., sensors) in the environment. The number of submodular function values is $2^{4}$. The order of set is defined as the number of selected sets. The number of $n^{th}$ order terms is $C_{n}^{N}$, where *N* is the total number of sets. Hence, the numbers of 0th, 1st, 2nd, 3rd, and 4th order terms are 1, 4, 6, 4, and 1, respectively. In case A, only sets 2 and 3 have sensing overlapping. Hence, only $f_{2,3}$ of the 2nd order terms is non-zero. There is no overlap between the third and fourth order sets, so the Fourier coefficients of 3rd and 4th orders are zero. Therefore, the number of non-zero coefficients in case A is 1+4+1=6. In case B, there is sensing overlapping between all sets, Hence the number of non-zero coefficients in case B is 1+4+6+4+1=16. This example demonstrates that utilizing the overlapping relationship can dramatically reduce the number of Fourier basis from $2^{N}$ to polynomial numbers if there are a few sensing overlaps between sets.

The major assumptions of SFSS are that if there are a few overlapping sets, the Fourier basis can be dramatically reduced. If most of the sets have sensing overlapping, the number of Fourier basis is close to $2^{N}$. Since the SFSS approach adopts Hadamard transform, this transform limits the possibility of Fourier basis selections. To solve this issue, finding another transform (e.g., deep neural networks) could lead to different sparsity in certain domains.

### 3.2. Learning Submodular Functions via Deep Compressed Sensing

The prior research of deep compressed sensing was for image or video applications [23,30,31,32], wherein the signal size is fixed. The challenge of deep compressed sensing techniques for submodular functions is that the networks must avoid processing the original signal since its size is $2^{N}$. Even if N=50, the networks cannot save the weighting parameters and compute the forward propagation. Hence, the key to design SDCS is to estimate the signal in the Fourier domain and then reconstruct the signal in the spatial domain.

The definition of learning submodular functions via deep compressed sensing is as follows:

**Definition** **2.** *Learning submodular functions in the Fourier domain:*

*Given a finite set S = {1,2,…,N}, submodular data (y), and corresponding set data (X), the learning coefficient of the submodular function in the Fouier domain (f) is: $\hat{f}=min_{f}||\Psi \left(X,f\right)-{y||}_{2}^{2}+{\lambda ||f||}_{1}$, where *Ψ* is a reconstruction function, λ is the parameter to tune the sparsity of f, $\left|\right|\cdot {\left|\right|}_{2}$ is the L2 norm, and $\left|\right|\cdot {\left|\right|}_{1}$ is the L1 norm.*

For example, the given data are N=3, y={0.3,0.4}, and X={0,0,1;0,1,0}. That means there are three sets. When the third set is selected (X={0,0,1}), its submodular value is 0.3 (y=0.3). When the second set is selected (X={0,1,0}), its submodular value is 0.4 (y=0.4). In fact, this is an ill-conditioned case, since the number of the unknown variable is $2^{3}$ and the number of measurements is 2. However, if the submodular function in the Fourier domain is sparse, it is possible to learn it in the Fourier domain first and then reconstruct it in the spatial domain. Hence, the goal is to find the submodular function in the Fourier domain (f) first through given *X* and *y*.

**Definition** **3.** *reconstruction of submodular functions in the spatial domain:*

*Given a finite set S = {1,2,…,N} and corresponding set data (X), the reconstruction of submodular functions in the spatial domain (y) is: y=Ψ(X,f).*

For example, N=3, f={0.7,0.1,0,0.2,0,0,0,0}, and X={0,0,1;0,1,0}. The submodular values can be reconstructed through the given *f* and Ψ function.

The major problems of learning and reconstruction of submodular functions are as follows: First, what is the Fourier transform (Θ), which makes submodular functions sparse? Second, what is the submodular function in the Fourier domain (*f*)? Third, how can one reconstruct the submodular function (*F*) through (f)? To solve these problems, this research proposes submodular deep compressed sensing (SDCS) to learn submodular functions. There are three stages of SDCS: transformation learning, Fourier coefficient learning, and reconstruction.

In the transformation learning stage (see Figure 2a), the goal is to train the autoencoder, which consists of encoder and decoder networks. The input data is the submodular data of a measurement set in different environments $\left(F_{m}^{1:M}\right)$, where *F* denotes submodular values, *m* denotes the size of measurements, and *M* denotes the number of the environments. Each submodular datum also encodes the set combination. The data connect to the L layers encoder $\left(\tilde{\theta }\right)$ and the encoder outputs to the Fourier coefficients (f). The Fourier coefficients (f) connect to the L layers decoder (θ) and the decoder outputs to the weighting networks (W). The combination data (X) are an *n* by *m* matrix. This matrix (X) and the weighting networks (W) output the estimated submodular values ${\hat{F}}_{m}^{1:M}$. Mathematically, the transformation is: ${\hat{F}}_{m}^{1:M}=W\left(X,\theta \left(f\right)\right)=\Psi \left(X,f\right)$, where Ψ is the reconstruction function, which reconstructs the submodular values given the corresponding *f* and *X*. The objective function of transformation learning stage is:

(1) $$\begin{array}{c} min_{\theta ,W}||\Psi \left(X,f\right)-F_{m}^{1:M}{\left|\right|}_{2}^{2} \end{array}$$

In the Fourier coefficient learning stage, the Fourier coefficients $\left(r_{t}\right)$ at time t connect to the decoder (θ). The decoded data (θ(f)) and the weighting networks (W) with combinational input (X) generate the predicted submodular data (y). The *y* is optimized via ADAM and updated as $r_{t+1}$. There are k phases in this stage. The loss function of this stage is:

(2) $$\begin{array}{c} min_{f}||\Psi \left(X,f\right)-{y||}_{2}^{2}+\lambda \left|f\right| \end{array}$$

In the reconstruction stages, the learned Fourier coefficients (f) are decoded by the decoder Θ. The decoded data Θ(f) and the weighting networks (W) with combinational input (X) generate the reconstruction data (y).

(3) $$\begin{array}{c} y=W(X,\Theta (f))=\Psi (X,f) \end{array}$$

## 4. SDCS Algorithm

There are three algorithms for learning and recovering submodular functions: transformation learning, Fourier coefficient learning, and reconstruction.

In the transformation learning stage (Algorithm 1), the transformation is an autoencoder based framework with fully connected neural networks (FCN) or convolutional neural networks (CNN). The training data are the submodular data from a measurement set ($F_{m}^{1:M}$), which include *m* data in different *M* environments and their corresponding set combinations (*X*). The weighting vector in the last layer of a decoder is the output of another weighting network (*W*) whose input is *X*. All weights in each layer of an autoencoder and *W* are initialized as a normal distribution. Lines 4–7 show one epoch of the training step. The number of epochs is 3000 and the batch size is 100. Line 5 is to do forward propagation from the batch data. Line 6 is to calculate the loss function (see Equation (1)), and adjust the model parameters via the Adam optimization. After training, all weights and biases are saved for learning Fourier coefficients.

In the Fourier coefficient learning stage (Algorithm 2), the goal is to learn *f* through training data. The training data are the submodular data from a measurement set ($F_{m}$). Notice that the measurement data are from the same environment to ensure the parametrical consistency of the neural networks. Line 4 is to run soft-thresholding for computing sparse *f* with the threshold (λ). Line 5 is to compute submodular function values based on current *f*. Line 6 is to update *f* according to data ($F_{m}$ and *X*) and Adam optimization (see Equation (2)). After training, the *f* is saved for the reconstruction of functions.

In the reconstruction stage (Algorithm 3), the goal is to predict *F* given any combination set (*X*). Line 3 shows that the neural networks process the set (X) and *f* to predict the submodular values. The major difference between compressed sensing for image data and submodular functions is that the data size of submodular functions is $2^{N}$. Hence, in the reconstruction step, it is infeasible to reconstruct all values of submodular functions. Algorithm 3 shows that the neural networks only predict submodular values according to the input set (*X*).
**Algorithm 1:** Transformation learning.1: Input: $F_{m}^{1:M}$, corresponding set combination *X*2: Initial weights in the encoder ($\tau_{e}^{0}$), decoder ($\tau_{d}^{0}$) and *W* ($\tau_{W}^{0}$)3: **for** e=1:epoch **do**4: **for** b=1:♯(batches) **do**5:  ${\left({\hat{F}}_{m}^{batch(b)}\right)}^{n}=W\left(X,\theta \left(\tilde{\theta }\left(F_{m}^{batch(b)},\tau_{e}^{n}\right),\tau_{d}^{n}\right),\tau_{W}^{n}\right)$6:  $\tau_{e}^{n+1}$, $\tau_{d}^{n+1}$, $\tau_{W}^{n+1}$ = AdamOptimizer($\left|\right|{\left({\hat{F}}_{m}^{batch(b)}\right)}^{n}-F_{m}^{batch(b)}{\left|\right|}_{2}^{2}$)7:  **end for**8: **end for**9: Save trained $\tau_{e}$, $\tau_{d}$, $\tau_{W}$
**Algorithm 2:** Fourier coefficient learning.1: Input: $F_{m}^{M_{i}}$, *X*, trained $\tau_{d}$, $\tau_{W}$, threshold λ2: Initial Fourier coefficients ($\gamma^{0}$)3: **for** e=1:epoch **do**4: $f^{n}=soft\left(\gamma^{n},\lambda \right)$5: ${\left({\hat{F}}_{m}^{M_{i}}\right)}^{n}=W\left(X,\theta \left(f^{n},\tau_{d}\right),\tau_{W}\right)$6: $\gamma^{n+1}$ = AdamOptimizer($\left|\right|{\left({\hat{F}}_{m}^{M_{i}}\right)}^{n}-F_{m}^{M_{i}}{\left|\right|}_{2}^{2}$)7: **end for**8: Save trained *f*
**Algorithm 3:** Reconstruction.1: Input: Set combinations *X*, Trained $f,\tau_{d},\tau_{W}$2: Output: submodular values (*F*) corresponding to input combinations (*X*)3: $F=W(X,\theta \left(f,\tau_{d}\right),\tau_{W})$

## 5. Experiments

**Figure 5:**
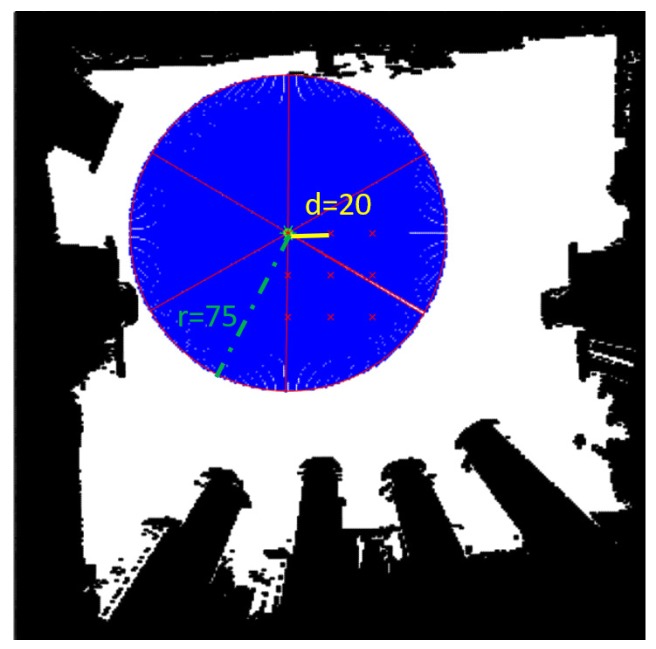
The subgoal configuration in the experiment. The red points represent the subgoal locations. The black and white grids represent the occupied and unoccupied grids, respectively. The blue areas and red lines represent the areas covered and field of view, respectively. The sensor-covered radius is 75 and the distance between subgoals is 20.

The goal of the experiment is to evaluate the learning performance of the proposed algorithm (SDCS) and compare with the prior work (SFSS) [3]. The adopted performance metrics are the mean error of estimated coverage, the results of the greedy algorithm, and the number of Fourier support (non-zero coefficients). For each approach, the number of all subgoals |S| is 54. The subgoal configuration (see Figure 5) illustrates the performances of three approaches in the experiments. Selecting the optimal solutions of three approaches is infeasible, since it needs to compute ${\left|54\right|}^{G}$ solutions where *G* is the number of selected subgoals. The coverage of selected subgoals of three approaches is compared with *G* = 15 in Section 5.3. Hence, the greedy algorithms are adopted for the three approaches to finding near-optimal solutions [7]. In Section 5.1 the experimental setup is described. In Section 5.2 the reconstruction results and sparsity of three approaches are compared. In Section 5.3, the greedy results of three approaches are compared. In Section 5.4, the computational time of three approaches is compared.

### 5.1. Experimental Setup

**Figure 6:**
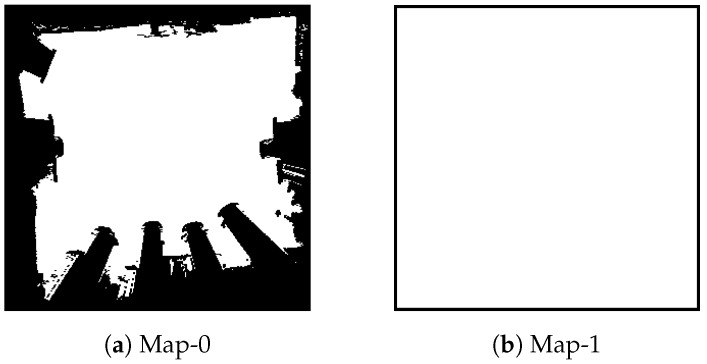
The experimental environments. The black and white grids represent the occupied and unoccupied grids, respectively. (**a**) A grid map built in a Lab environment. (**b**) A grid map without any obstacles.

The maps and subgoals configurations are as follows: The experimental environments are 300×300 grid maps (see Figure 6). The Map-1 is adopted for training Θ. Map-0 is adopted for training *f* and testing reconstruction results. The subgoal configuration is shown in Figure 5. The range and field of view for each subgoal are 75 and $60^{\circ }$, respectively. There are nine subgoals with six directions for each subgoal (i.e., there are 54(9×6) subgoals). The distance of two adjacent subgoals is 20.

**Figure 7:**
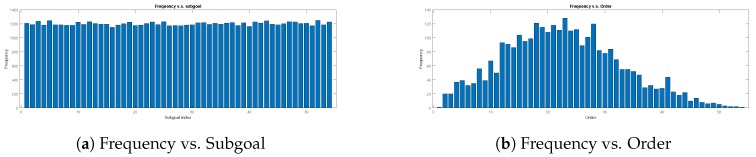
The distribution of 3000 test data. The sugoal is chosen uniformly, and the order is normal.

**Figure 8:**
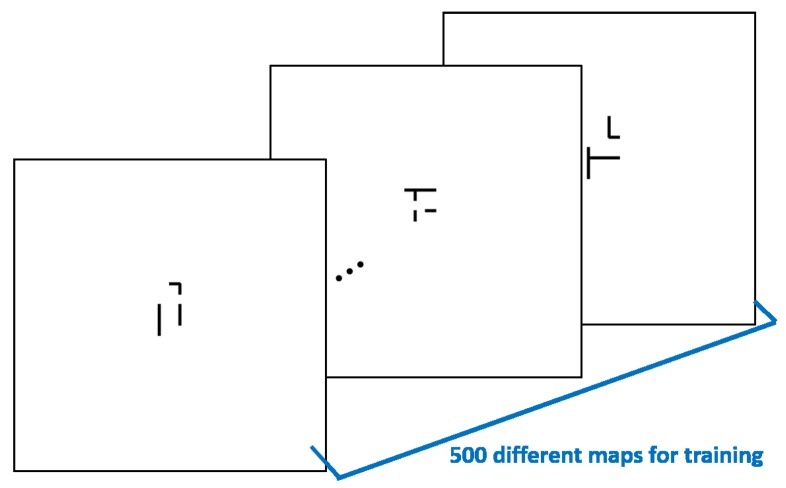
The training data for the transformation stage. The black areas represent different obstacles in each map.

The training data are collected by 3000 set combinations, which are randomly selected. The distributions of each subgoal’s frequency and the order of the combinations are shown in Figure 7. There are 500 different training maps (see Figure 8) which are randomly generated obstacles in Map-1. The input dimension of the transformation learning stage is 100×3000 in every batch.

**Figure 9:**
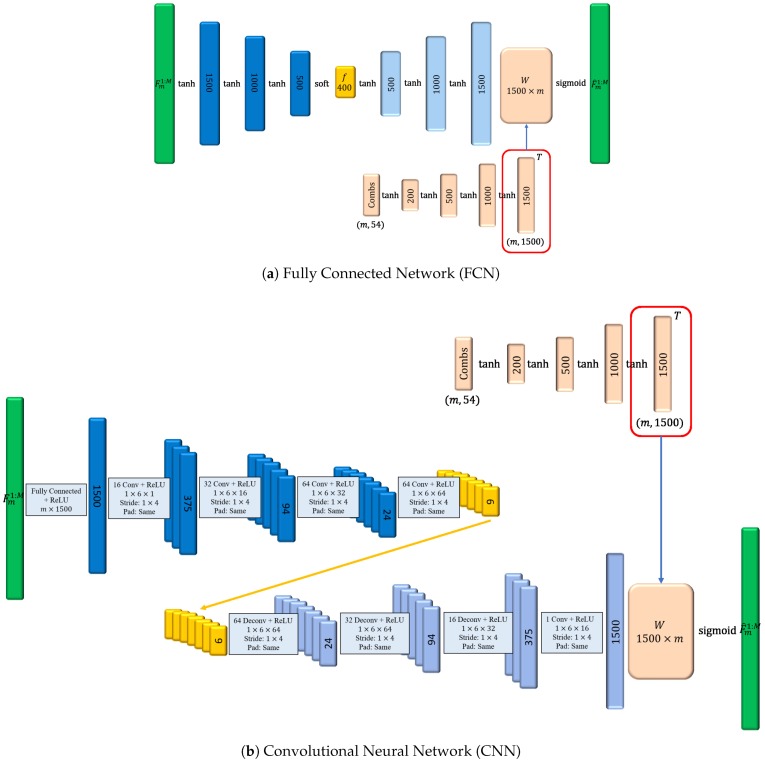
The frameworks of the transformation learning. This stage includes an autoencoder, weighting networks (W), and combinational networks.

Since the network structures of the Fourier coefficient learning and reconstructions stages are similar to that of the transformation learning stage, the network structures of the transformation learning stage are explained as follows (see Figure 9): This stage includes an autoencoder, weighting networks (*W*), and combinational networks. The autoencoder is implemented by two network structures, a fully connected neural network (FCN) and a convolutional neural network (CNN).

The structure of the FCN autoencoder is as follows (see Figure 9a): In the encoder, there are three hidden layers, and the dimensions of each layer are 1500, 1000, and 500, respectively. The dimension of transformed coefficients (*f*) is 400. In the decoder, there are three hidden layers but the dimensions are converse. The activation functions of each layer are hyperbolic tangents except the output layers of the encoder and decoder. The output layer’s activation function of the encoder is a soft-threshold function with λ=0.01, while that of the decoder is a sigmoid function. In the weighting network (*W*) of the decoder output layer, the dimensions are 200, 500, and 1000. The output dimension is the same as the output layer of the decoder. The transpose of this part’s output (see “*T*” in Figure 9) is the weights of the output layer in the decoder.

The structure of the CNN autoencoder is as follows (see Figure 9b): The first layer and the output layer are fully connected. There are four convolution layers in the encoder and four deconvolution layers in the decoder. In each hidden layer, the filter size of a channel is 1 × 6 and the stride is 1 × 4. The activation functions in the output layer of encoder and decoder are soft-threshold functions with λ=0.01 and sigmoid function, respectively. The numbers of filters in each layer in the encoder are 16, 32, 64, and 64.

**Figure 10:**
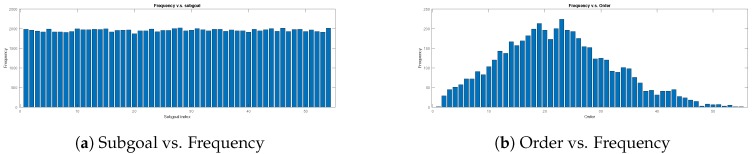
The distribution of 5000 test data. The subgoals are chosen uniformly, and the order of subgoals is normal.

In the experiment, all of the weights of each layer are initialized in a truncated normal distribution (∼N(0,0.07)) and the biases are set as 0.05. The epoch of the transformation training stage is 3000 while that of the Fourier coefficient training stage is 1000. The prediction errors are computed by 5000 different set combinations (Figure 10) in Map-0.

SDCS is implemented via Tensorflow in Python while SFSS is implemented via sklearn package in Python. All the experiments are performed on a workstation with Intel Core i7-8700k CPU and GTX2080Ti GPU.

### 5.2. Reconstruction Results vs. Sparsity

**Figure 11:**
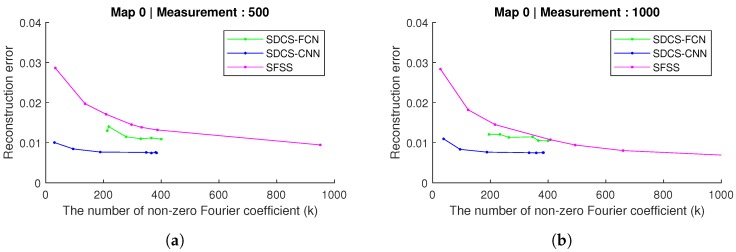
When the distances of two adjacent subgoals are 20, the figure shows the k-sparse in transferred coefficient vs. mean error between reconstructed result and ground truth in Map-0 and two different numbers of measurements. The reconstruction resulted in 500 and 1000 measurements in Map-0 (Figure 6a).

In this experiment, the distance between two adjacent subgoals is 20, and the number of Fourier basis *b* of SFSS is 9852; it is decided by the algorithms in [3]. The number of bases (|f|) of SDCS-CNN and SDCS-FCN is 384 and 400, respectively. The different thresholds (λ) are tested to get the reconstruction in the Fourier coefficient learning stage. The thresholds are set as follows: SDCS-CNN: [$1\times 10^{-5}$, 0.001, 0.005, 0.01, 0.05, 0.1, 0.15]; SDCS-FCN: [$1\times 10^{-5}$, 0.0005, 0.001, 0.005, 0.01, 0.02]; SFSS: [$1\times 10^{-5}$, 0.001, 0.0015, 0.002, 0.0035, 0.005, 0.01]. As Figure 11 shows, when the number of non-zero elements in *f* is lower than 400, the mean error of SDCS-FCN and SDCS-CNN is lower than that of SFSS. These experiments demonstrate that SDCS approach is able to reconstruct submodular functions using fewer Fourier bases than the SFSS approach does.

### 5.3. Greedy Results vs. Sparsity

**Figure 12:**
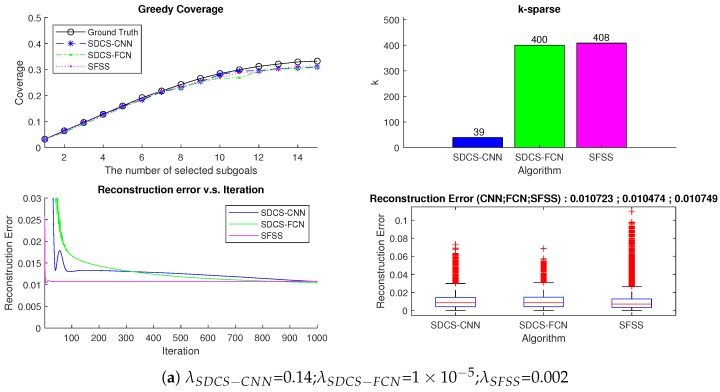

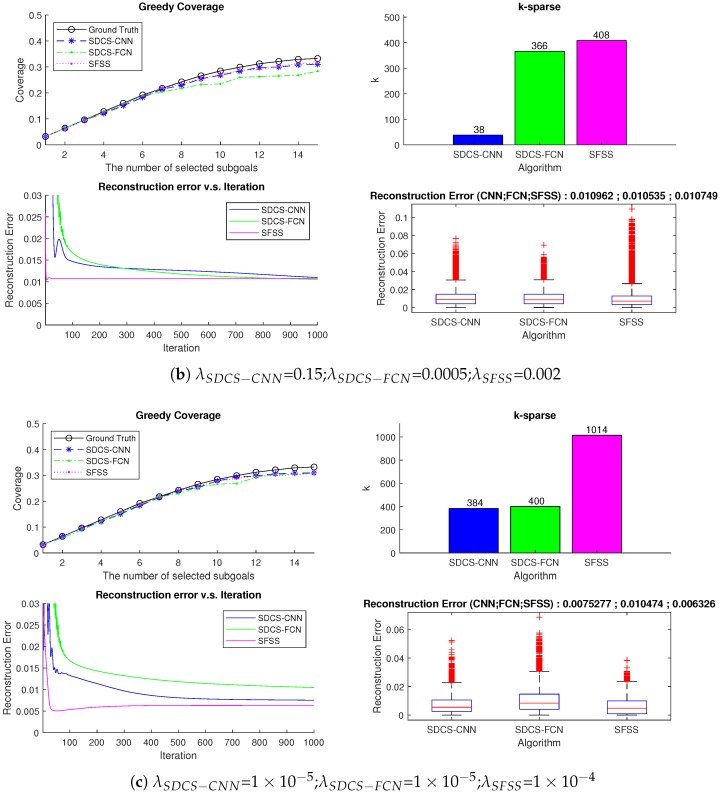
Comparison of results of three approaches.

The reconstruction results having lower errors does not mean that the coverage of selected subgoals (so called greedy results) is higher. The *k* is defined as the number of the non-zero Fourier coefficients. The test data are 1000 measurements in Map-0 (see Figure 12). The reconstruction error and greedy results of three approaches are further compared with different λ parameters.

**Figure 13:**
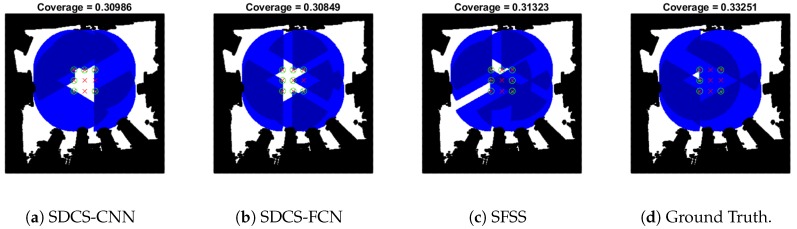
Coverage area is shown in Map-0 with Figure 12a. The black and white grids represent the occupied and unoccupied grids, respectively. The blue grids represent the coverage area of the sensor. The grid being dark blue means that this grid is covered by at least two sensors. The green circle and red cross represent the subgoal being selected or not.

As Figure 12a shown, when the reconstruction error of three approaches is around 0.01, their greedy results are similar. The number of non-zero elements of SDCS-CNN is just 38, which is smaller than that of SDCS-FCN and SFSS. The coverage area in the Map-0 of each approach is shown in Figure 13. The greedy results of SDCS approach are similar to those of SFSS, while the *k* of SDCS is lower than that of SFSS (38 vs. 408).

**Figure 14:**
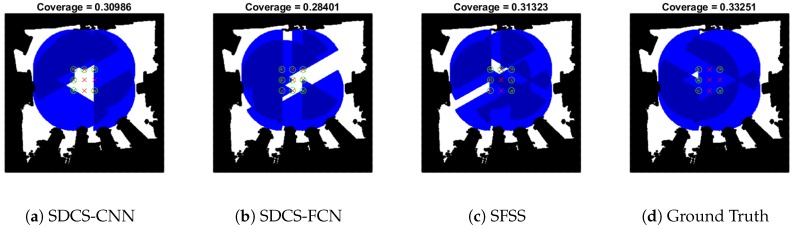
Coverage area is shown in Map-0 with Figure 12b.

As Figure 12b shows, the reconstruction’s mean error is still around 0.01 when the λ of SDCS-CNN increases from 0.14 to 0.15 and λ of SDCS-FCN increases from $1\times 10^{-5}$ to 0.0005, but SDCS-FCN greedy results are worse. The coverage area is shown in Figure 14. The experiments in Figure 12a,b demonstrate that the greedy results of SDCS-CNN are more stable than that of SDCS-FCN. In other words, CNN is more suitable for SDCS framework than FCN.

**Figure 15:**
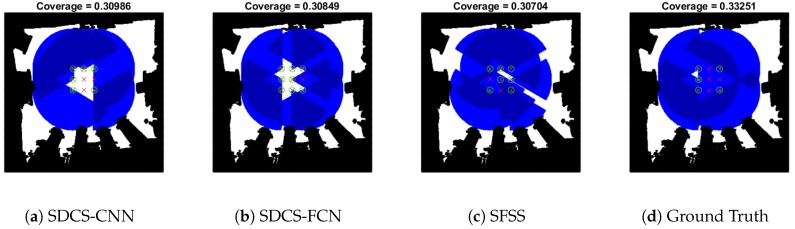
Coverage area is shown in Map-0 with Figure 12c.

As Figure 12c shows, the performances of the three approaches with the smallest λ values in Section 5.2 were compared. SFSS has the lowest reconstruction mean error, but the *k* is the biggest (1014). Although the reconstruction error of SDCS-CNN is more than that of SFSS, the greedy results of three approaches are similar. This experiment demonstrates that SFSS can be replaced by SDCS-CNN (see Figure 15).

### 5.4. Computational Time

**Figure 16:**
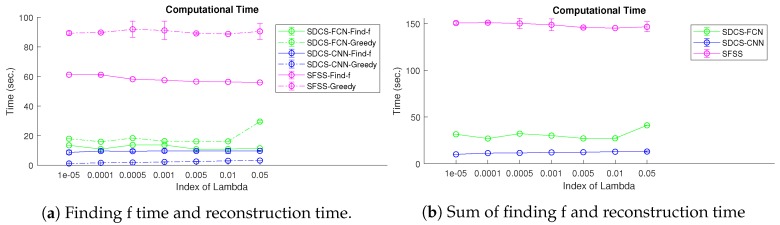
Computational time of three approaches.

In this experiment, the execution time for the three approaches is calculated. All approaches are implemented via Python on a workstation. The time of training transform and collecting the training data in SDCS and the time of finding the basis in SFSS are not considered. The execution time includes learning the Fourier coefficients and reconstruction. We calculate the time to find 30 subgoals using the greedy algorithm to represent the reconstruction time. There are seven different thresholds ($1\times 10^{-5}$, 0.0001, 0.0005, 0.001, 0.005, 0.01, 0.05). For each approach with each threshold, we compute the execution time 10 times and calculate the mean and the variance of them. Figure 16 shows that SFSS needs more time to find the coefficient and do reconstruction, since the Ψ matrix is computed by sampling and basis combination where the basis is 9852 in this experiment. Hence, SDCS is faster than SFSS when the Fourier basis is large. This experiment shows that SDCS is more efficient than SFSS when most of the sets’ sensing areas are overlapped.

## 6. Conclusions

In this paper, a deep compressed sensing approach is proposed to learn submodular functions. The contributions of the research are as follows: First, the proposed algorithm is able to learn submodular functions via deep compressed sensing techniques. Second, the nonlinear transformation network can be learned through different data. In other words, the network automatically finds the Fourier domain, which generates the sparse coefficients (f). Third, the experiments demonstrate that the proposed algorithm is more accurate than the benchmark approach when the number of Fourier bases is 200∼400.

The future work of this research is as follows: First, spatial search problems are potential applications of submodular functions. If the proposed algorithm is applied to spatial search, it could improve the learning efficiency. Second, in most of the compressed sensing approaches, the transformation functions are given. This research shows a way to learn the transformation functions through data. Finding optimal Fourier bases (e.g., a minimal number of Fourier bases) is a potential research topic. Finally, since humans are able to search target environments efficiently, exploring how humans solve search problems is another way to construct neural networks. A promising approach is to let human subjects remotely control the robot searching the environment. Then, the networks could be learned via deep inverse reinforcement learning.
